# Snowflake Vitreoretinal Degeneration (SVD) Mutation R162W Provides New Insights into Kir7.1 Ion Channel Structure and Function

**DOI:** 10.1371/journal.pone.0071744

**Published:** 2013-08-19

**Authors:** Bikash R. Pattnaik, Sara Tokarz, Matti P. Asuma, Tyler Schroeder, Anil Sharma, Julie C. Mitchell, Albert O. Edwards, De-Ann M. Pillers

**Affiliations:** 1 Department of Pediatrics, University of Wisconsin, Madison, Wisconsin, United States of America; 2 Department of Ophthalmology and Visual Sciences, University of Wisconsin, Madison, Wisconsin, United States of America; 3 McPherson Eye Research Institute, University of Wisconsin, Madison, Wisconsin, United States of America; 4 Department of Biochemistry, University of Wisconsin, Madison, Wisconsin, United States of America; 5 Department of Experimental Pathology, Mayo Clinic, Rochester, Minnesota, United States of America; 6 Institute for Molecular Biology, University of Oregon, and Oregon Retina, Eugene, Oregon, United States of America; Tohoku University, Japan

## Abstract

Snowflake Vitreoretinal Degeneration (SVD) is associated with the R162W mutation of the Kir7.1 inwardly-rectifying potassium channel. Kir7.1 is found at the apical membrane of Retinal Pigment Epithelial (RPE) cells, adjacent to the photoreceptor neurons. The SVD phenotype ranges from RPE degeneration to an abnormal b-wave to a liquid vitreous. We sought to determine how this mutation alters the structure and function of the human Kir7.1 channel. In this study, we expressed a Kir7.1 construct with the R162W mutation in CHO cells to evaluate function of the ion channel. Compared to the wild-type protein, the mutant protein exhibited a non-functional Kir channel that resulted in depolarization of the resting membrane potential. Upon co-expression with wild-type Kir7.1, R162W mutant showed a reduction of I_Kir7.1_ and positive shift in ‘0’ current potential. Homology modeling based on the structure of a bacterial Kir channel protein suggested that the effect of R162W mutation is a result of loss of hydrogen bonding by the regulatory lipid binding domain of the cytoplasmic structure.

## Introduction

A point mutation in *KCNJ13*, the gene encoding the protein subunits of the inwardly rectifying potassium channel Kir7.1, is associated with an autosomal dominant Snowflake Vitreoretinal Degeneration (SVD) [1]. Kir7.1 is a member of the Kir channel family and is expressed predominantly in the retina [2], [3]. Classic SVD features include snowflake-like deposits in the retina, focal degeneration of the retinal pigment epithelium (RPE), moderate myopia, an optically empty vitreous, and a reduced scotopic b-wave. Patients with SVD also develop corneal guttae indistinguishable from those found in typical Fuchs corneal dystrophy [4], [5].

Trans-membrane ion flux via a variety of ion channels, transporters, and pumps supports epithelial fluid transport and maintains diverse physiologic functions [6]. Within the retina, the Kir7.1 channel is found in the apical processes of the RPE at the interface with the photoreceptor outer-segment [7]. Kir7.1 allows passage of K^+^ into and out of the RPE cells, thereby contributing to ion homeostasis in the sub-retinal space [8]. The Kir 7.1 channel thus supports many aspects of RPE physiology, including maintenance of the membrane potential, recycling of potassium ions, and directional fluid transport where the trans-RPE ion flux is crucial to maintaining the communication between the RPE and retina that is essential for vision [9], [10]. Kir channels are comprised of four protein subunits, with each containing an N-terminal cytoplasmic loop, two trans-membrane segments, one selectivity P-loop, and a cytoplasmic C-terminal regulatory domain [2], [11]. The SVD mutation R162W is located within a conserved cluster of C-terminal basic residues. In the rat Kir7.1 mutant model, Hejtmancik and colleagues showed that switching the arginine (R) residue at position 162 to a tryptophan (W) results in a non–selective, leaky channel [1] with a reversal potential near zero. In addition to the point mutation at position 162, the rat clone had an additional upstream mutation: A139S. The Kir7.1 phenotype described in the rat model is complicated by this second mutation and thus may not be an ideal model for SVD pathogenesis. Our work differs from the earlier publication in that we have excluded any potential direct effects from the second mutation, or any interaction between the two mutations which could lead the rat Kir7.1 mutant channel to behave differently *in vivo* and *in vitro* than its human counterpart. It is also possible that subtle differences in the structure-function relationship exists between rat and human Kir channel.

How an RPE apical membrane channelopathy results in a spectrum of eye pathology that involves the RPE, retina, vitreous, and the cornea is unclear. Likewise, little is known about how the function of the mutant human Kir7.1 channel relates to the phenotype associated with SVD. In this paper, we expressed both wild-type and R162W mutant human Kir7.1 channels in CHO and HEK mammalian cells. Using the whole-cell configuration of the patch-clamp technique, we characterized the functional differences between the wild-type and the mutant channels. Fluorescence fusion enabled visualization of membrane expression of the wild-type and mutant proteins. Structural alterations due to this mutation were analyzed by molecular modeling.

## Materials and Methods

### Ethics Statement

All animal studies were in compliance with the requirements of the ARVO Statement for the Use of Animals in Ophthalmic and Vision Research (http://www.arvo.org/policies/statement_for_the_use_of_animals_in_ophthalmic_and_visual_research/). All experimental protocols using rodents were reviewed and approved by the Animal Care and Use Committee of the University of Wisconsin-Madison.

### Reagents

All chemical reagents were obtained from Fisher Scientific (Pittsburgh, PA) or Sigma (St. Louis, MO).

### Cell Culture

CHO-K1 cells (ATCC, Manassas, VA) were chosen because they do not have endogenous expression of Kir channels [12]. Cells were maintained in HAMs F-12 medium supplemented with 5% FBS and 1% penicillin/streptomycin at 37°C in a humidified 5% CO_2_ incubator [12]. The media was changed at 2–3 da intervals and the cells were used within 15 passages. HEK-293, another established cell line frequently used to study heterologous expression of ion-channels [13], was cultured in EMEM (Eagle’s Minimum Essential Medium) containing 10% FBS and 1% penicillin/streptomycin, as above. The media was changed every 2–3 days and the cells were split weekly.

### Plasmid Vectors and Transfection

To express the Kir7.1 channel and to study its electrophysiology, the full length cDNA clone of human KCNJ13 (NM_002242.2) cloned into the pCMV6-XL5 eukaryotic expression vector was obtained from ORIGENE (Cat no.TC128213, Rockville, MD). To generate an N-terminal Green Fluorescence Protein (GFP) construct, we amplified the KCNJ13 open reading frame flanked by the EcoR1 and BamH1 restriction sites using primer pairs:

Forward-(5′-GCTTCGAATTCCGACAGCAGTAATTGC-3′), and Reverse-(5′-ATCCGGTGGATCCTTATTCTGTCAGTCC-3′).

The amplified product was cloned into the multiple cloning site (MCS) of vector pEGFP-C1 (Clontech Laboratories Mountain View, CA). The mutant clone was generated by introducing the mutation (484 C>T) into the wild-type KCNJ13 (NM_002242.2) clone using the “Quick-Change Site-Directed Mutagenesis Kit” from Stratagene (Agilent Technologies Inc., Santa Clara, CA). We generated an N-terminal mCherry fusion construct using the EcoR1 and BamH1 restriction cloning sites in the MCS of the vector pmCherry-C1 (Clontech Laboratories Inc. Mountain View, CA), as described above for the wild-type fusion construct. mCherry is a monomeric protein. By creating a tagged mutant protein, we were able to determine the co-localization of GFP and mCherry proteins, corresponding to the wild-type and mutant Kir7.1 channels, respectively. We studied one wild-type (pEGFP-hKir7.1) and two mutant fusion plasmids (pEGFP-R162W and pmCherry-R162W). The composition of each construct was confirmed by sequencing.

CHO-K1 cells (400,000 cells per 35 mm culture dish) were transfected using either TransIT-CHO reagents (Mirus Bio LLC, Madison, WI) or by nucleofection (Lonza, Walkersville, MA) per the manufacturers’ guidelines. HEK-293 cells were transfected by nucleofection. We used the following plasmid combinations for transfections: 1) pEGFP-hKir7.1 plus pmCherry, or 2) pmCherry-R162W plus pEGFP, or 3) pEGFP-hKir7.1 plus pmCherry-R162W. Two µg of plasmid DNA (1 µg per plasmid) was transfected per 35 mm culture dish. Cells were trypsinized after 24 hrs of transfection, plated on 12 mm #1 glass cover slips and studied within 24 to 72 hrs of transfection.

### Electrophysiological Recording

Kir channel currents were recorded by whole-cell patch clamp electrophysiology at room temperature using an Axopatch 200B amplifier (Molecular Devices, Sunnyvale, CA) and the Digidata 1440A data acquisition system (Molecular Devices, Sunnyvale, CA). The data were low-pass filtered at 1 kHz, and digitized at 0.5 kHz. The cell-covered glass coverslips were placed in the bottom of a 13 mm chamber mounted on the fixed stage of a Nikon FN-1 microscope and continuously perfused using the following bath solution (HR), in mM: 135 NaCl, 5 KCl, 10 HEPES, 10 glucose, 1.8 CaCl_2,_ and 1 MgCl_2_. The pH was adjusted to 7.4 using NaOH. We substituted 20 mM Cs^+^ for Na^+^ in the bath solution to block Kir7.1 channel current. To determine the selectivity of the Kir7.1 channels, Na^+^ salt in the Ringer’s solution was replaced with either 135 mM K^+^ or Rb^+^ in the bath. Rapid exchange of the solution in the recording chamber was achieved using a gravity-feed system controlled by computer-programmed valves (Pattnaik, unpublished).

Patch pipettes were made from borosilicate glass (BF150-117-10, Sutter Instruments, Novato, CA) using a horizontal pipette puller (P-1000, Sutter Instruments, Novato, CA) and fire polished (MF-830, Narishige, Tokyo, Japan). The recording electrodes, when filled with (in mM) 30 KCl, 83 K-gluconate, 10 HEPES, 5.5 EGTA, 0.5 CaCl_2_, 4 Mg-ATP, and 0.1 GTP, and adjusted to pH 7.2 using KOH, measured a tip resistance of between 2.5–3.5 MΩ. A saturated salt agar bridge was used as a reference electrode. Whole cell capacitance and series resistance were monitored and compensated throughout the recording. The current was monitored either using a linear voltage ramp from −160 mV to +40 mV or a voltage step series between −160 mV to +50 mV. In between recordings, the cell was held at −10 mV. In each of our recordings, we use the term inward-current to refer to an inward K^+^ ion flow. All recordings were performed at room temperature.

### Antibodies

Kir7.1 polyclonal antibodies were the generous gift of Dr. Bret Hughes (University of Michigan, Ann Arbor). Monoclonal cone antibody 7G6 was kindly provided by Dr. Peter MacLeish (Morehouse School of Medicine, Atlanta, GA). Kir7.1 polyclonal antibodies from Osenses (Osenses Pty Ltd., Keswick, Australia) and β-actin monoclonal antibody from LI-COR Biosciences (Lincoln, NE) were also used.

### Immunofluorescence Staining

*Macaca mulatta* eyes were obtained from the Wisconsin National Primate Research Center within 1 h of being euthanized for other unrelated studies. The anterior segment lens and vitreous were surgically removed. The eye cup was fixed in 4% paraformaldehyde and 1 cm^2^ pieces were embedded in OCT blocks. Ten µm frozen tissue sections were collected on charged slides and frozen at −80°C until use. Sections were thawed at room temperature and rehydrated in PBS before performing immunohistochemistry. After blocking with 5% goat serum and permeabilizing cells with 0.2% Triton X-100, the slides/coverslips were stained by incubating with the appropriate primary antibodies in blocking buffer overnight at 4°C. The samples were washed three times in PBS followed by exposure to fluorophore-conjugated secondary antibodies for 2 h at room temperature. The slides/coverslips were washed and mounted using Fluoromount (Sigma-Aldrich Corp., St. Louis, MO) and dried. All fluorescence images were acquired using a Nikon FN1 epifluorescence microscope and analyzed using NIS elements (Nikon, Melville, NY). In the case of the XYZ scan mode, a sequence of XY frames was obtained at 0.1 µm intervals in the Z-thickness.

### Live-cell Imaging

We used NucBlue™ (Life Technologies, NY) for nuclear staining and the ER-ID™ Red assay kit (Enzo Life Sciences, PA) for staining the endoplasmic reticulum. A day after nucleofection (Lonza, Germany), cells on the cover slip were incubated in a solution containing 1 µl each of ER-ID and Hoechst in 1X Assay buffer (ER-ID kit) and incubated for 30 minutes at 37°C in dark. The cells were washed one time in 1X Assay (ER-ID kit) buffer and mounted on an imaging chamber (Quick release magnetic chambers, Warner Instruments, CT). Cells were imaged using a 60X water immersion objective (Plan Apo 60X water, NA 1.0) in Ringer’s solution. Off-line data analyses were performed using NIS-Elements (Nikon USA, NY) and the fluorescence intensities were determined by line scan function.

### Western Blot

Whole cell lysates from transfected cells were prepared using RIPA buffer (150 mM NaCl, 1.0% NP-40, 0.5% DOC, 0.1% SDS, 50 mM Tris pH 8.0). Lysates were centrifuged to separate the soluble extract from the insoluble and cell debris portions. The soluble lysates were separated by 4–12% SDS-PAGE gel electrophoresis and transferred to Immobilon-FL PVDF membranes (Millipore, Billerica, MA). Membranes were blocked with Odyssey Blocking buffer: PBS with 0.2% Tween 20 (LI-COR Biosciences, Lincoln, NE). After transferring the proteins to the membrane, we visualized the protein of interest by immunostaining the Western blots with monoclonal anti-Kir7.1 (1∶250 dilution) or β-actin antibodies (1∶8000). Primary antibodies were diluted 1∶1 with Odyssey Blocking buffer. Primary antibodies were detected with secondary antibodies labeled with the near-infrared fluorescent dyes IRDye800 and IRDye700 (LI-COR Biosciences, Lincoln, NE) to allow two-color imaging and band overlay. Secondary antibodies were diluted 1∶15,000 in 1∶1 Odyssey Blocking buffer: PBS, 0.2% Tween 20. Proteins were visualized by scanning the blots with a LI-COR Odyssey Infrared Imager (Lincoln, NE). Kir7.1 and β-actin protein levels were quantified using the LI-COR Odyssey Infrared Imager software Version 1.2. Total Kir7.1 wild-type and mutant protein levels were normalized to β-actin and then ratios of Kir7.1 wild-type, R162W mutant, and Kir7.1 wild-type plus R162 mutant were determined. Experiments were performed in triplicate and the SEM determined.

### Molecular Modeling

A comparative three-dimensional model of the Kir7.1 protein was generated using the crystal structure of the Kir2.2 channel as a template (Protein Database accession number 3SPI) [14]. This structure represents the general Kir channel membrane configuration in association with PIP_2_ molecules. Similarly, crystal structure 3SPG served as a template for generating the model of mutant Kir7.1R162W protein structure with PIP_2_ [14]. These structures were solved by automated comparative protein modeling using MODELLER (http://salilab.org/modeller/) [15]. An advantage of using MODELLER in this context is that it can model all four monomers simultaneously, resulting in a more realistic model for the tetrameric assembly.

### Data Analysis and Statistical Methods

Data are represented as mean ± SEM from more than 5 observations. All data acquired using pClamp-10 were analyzed by Clampfit (Molecular Devices, Sunnyvale, CA) and the results were imported to Excel (Microsoft) for statistical analysis. We used the Student’s t test to determine statistical significance as defined by P-values <0.05. Data are represented as mean ± SEM, unless otherwise stated. Graphs and figures were made using Origin (Origin Lab, MA) and PowerPoint (Microsoft).

## Results

### Kir7.1 Protein Localization in the Primate Retina

**Figure 1:**
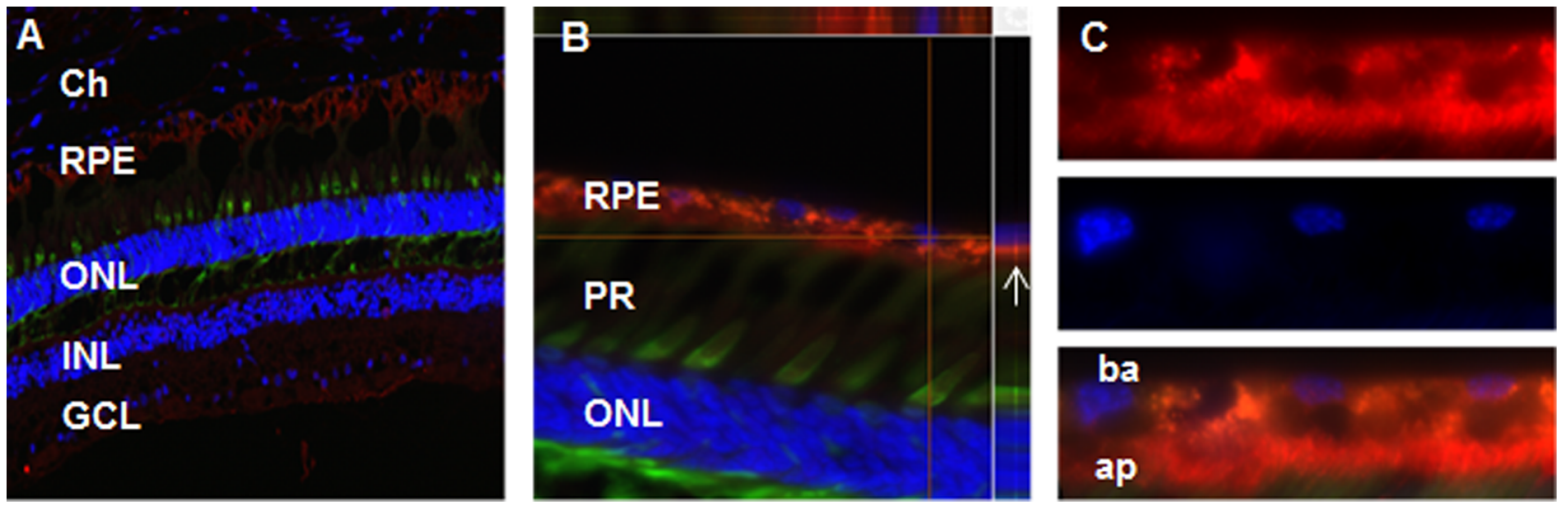
Kir7.1 channels are localized to the apical processes of adult rhesus RPE cells. (**A**) Low-magnification (10X, NA 0.30) view of a frozen section showing Kir7.1 immunopositive staining of the RPE cell layer. Kir7.1 (red) was detected in the RPE cell layer but was not detected in the neural retina. Cone photoreceptors (PR) are immunopositive for cone arrestin (green). Nuclear staining (blue) shows an intact retinal structure. (**B**) Images acquired using a 60X objective (60X water, NA 1.0) show the magnified outer nuclear layer (ONL) and the RPE cell layer. Z-stack images of the retina were obtained to generate a three-dimensional reconstructed image. The image above the top white line (**B**) is the optical cross section at the horizontal cursor, and the image on the right side of vertical white line is the optical cross section of the vertical cursor. Note the apical localization of the red fluorescence signal (arrow) which is present anterior to the RPE nuclear staining. (**C**) Higher magnification image (100X Oil, NA 1.4) showing 4 adjacent RPE cells illustrating the distribution of Kir7.1 immunostaining at the apical processes (upper panel). The middle panel shows the position of the RPE cell nuclei (DAPI) and the lower panel is a superimposed image of Kir7.1 and DAPI nuclear staining. Abbreviations used are: choroid (Ch), retina pigment epithelium (RPE), outer nuclear layer (ONL), inner nuclear layer (INL), ganglion cell layer (GCL), photoreceptors (PR), basal side (ba), and apical side (ap).

The retinal and vitreal phenotypes observed in SVD patients suggest that Kir7.1 plays an important role in the retina. Whether or not Kir7.1 is actually present in the retina is the subject of debate, although Kir7.1 expression has been demonstrated in the RPE apical processes [7], [16], [17]. To confirm the localization of Kir7.1 to the primate retina, we performed immunohistochemistry on RPE and retinal frozen sections from fetal and adult human tissues and adult monkey tissues. Adult monkey retina sections are shown in Fig. 1. Kir7.1 staining (red), using a well-characterized polyclonal antibody [7], was observed in a prominent monolayer in the posterior retina, mainly in the RPE cell layer. At a magnification of 100X (Fig. 1A), Kir7.1 staining was observed near the tip of cone photoreceptor outer segments, consistent with expression in the RPE. Confirmation of staining specific to the RPE cell apical process came from a subsequent higher magnification image (Figs. 1B 600X and C 1000X), and image reconstruction using Z-stack optical sections (Fig. 1B). Localization of Kir7.1 channels to the RPE was also found in both adult [18] and fetal human tissue sections (data not shown). The prominent localization of the Kir7.1 channel to the RPE apical processes supports a role for Kir7.1 channels in potassium homeostasis in the sub-retinal space. Our results using primate retina are consistent with the previous reports in the rat and human model that Kir7.1 localizes to RPE apical processes [16], [18]. None of the approaches have demonstrated any specific staining of Kir7.1 within the neural retina, however [7], [16], [17], [19].

### Altered Function of Human Kir7.1R162W Channel

**Figure 2:**
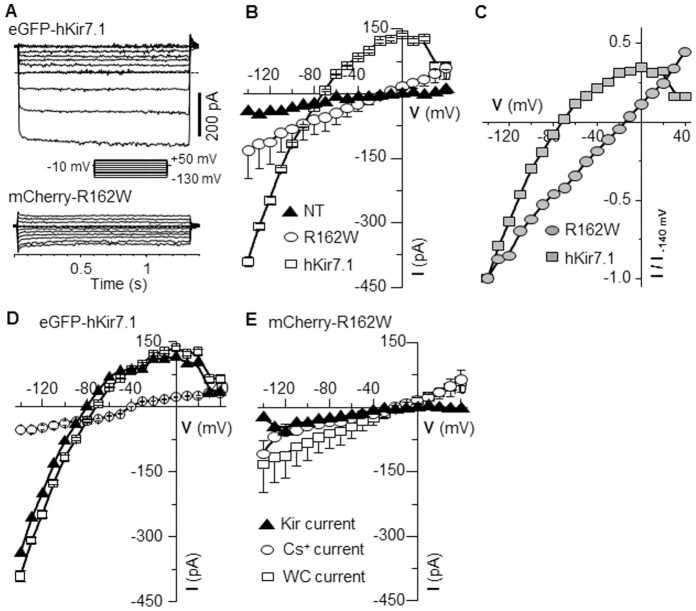
Kir7.1 R162W channels display altered function. (**A**) Representative recordings showing current responses to voltage pulses of 1.5 seconds. Hyperpolarizing potentials yield large inward currents and small outward currents due to the hKir7.1 channel (upper panel) whereas amplitudes of the outward and inward current were of similar magnitude for R162W channels (lower panel). The horizontal dashed line represents the zero current line. The voltage pulse protocol is indicated in the inset. (**B**) Average current amplitude vs. applied voltage (I–V) plot from non-transfected (NT-: closed triangles), pmCherry-R162W (R162W: open circles) and pEGFP-hKir7.1 (hKir7.1: open squares) transfected cells. The responses were averaged from at least 5 experiments. (**C**) Normalized currents (I/I_-150__mV_) reflecting a clear positive shift in the zero current potential (square-hKir7.1 and circle-R162W). (**D**) I–V plot from cells transfected with hKir7.1 channels showing whole cell current (open squares), and current in the presence of 20 mM Cs^+^ (open circle). The Kir current (closed triangles) is the Cs^+^ sensitive component that is obtained by mathematical subtraction (square minus circle). (**E**) I–V plot from cells transfected with pmCherry-R162W with symbols as in D. Error bars represent ± SEM.

To compare the biophysical properties of the hKir7.1 wild-type and R162W mutant channels, we transfected cells with the plasmids and recorded the resulting current in the whole cell mode. Representative current recordings are shown in Fig. 2. When pEGFP-hKir7.1 expressing cells were exposed to 1300 msec step-voltage pulses of various amplitudes (−150 to +50 mV) from a holding potential of −10 mV, the cells responded with an increase in current amplitude strictly in the hyperpolarizing direction. As shown in Fig. 2A (upper panel), only hyperpolarizing pulses registered a large inward current. In the representative cell, the current amplitude measured ∼ 400 pA in response to a −150 mV hyperpolarizing voltage pulse. The current activation was instantaneous without any time-dependent desensitization over the duration of voltage pulse, as is expected for Kir7.1 channels. Kir7.1 channels show a voltage-dependent reduction in the outward current amplitude [20], [21]. The outward current via the hKir7.1 channel was consistently reduced as the cell was depolarized. As noted in the figure, the current amplitude measured at a holding potential of −10 mV was positive (Note positive current as outward current compared to the dotted 0 current line in Fig. 2A) and current responses to a −70 mV pulse in Fig. 2A, upper trace lined with the 0 current line, indicating reversal potential of the channel. Using a similar protocol to that described above for pEGFP-hKir7.1, the current traces recorded from pmCherry-R162W plasmid-expressing cells were different from what is expected for Kir7.1 channel currents. Current amplitudes measured at −140 mV were less than 25% (∼ 98 pA) of the hKir7.1 channel current. The cell responded to depolarizing or hyperpolarizing step potentials with similar amplitudes of the outward and inward currents, respectively (Fig. 2A lower panel). The transfection efficiency was measured by counting fluorescence-positive cells and showed no difference between hKir7.1 GFP and R162W mCherry expressing cells (36±6% versus 33±8%).

To simplify the comparison of current amplitude from transfected cells (shown in Fig. 2A), recordings were averaged to display I–V relationship plots. The I–V plot for hKir7.1 transfected cells (Fig. 2B, square trace) showed a prominent inwardly rectifying current with little outward current, representing a reduction in slope conductance in response to depolarizing test potentials. The average current measured at −140 mV was 391±12 pA (n = 18) of inward current. The average current density was 28.5±2 pA/pF. The measured slope conductance was 5.4 nS for currents between −50 mV to −130 mV, and 1.3 nS for currents between −30 to +10 mV. This inwardly rectifying current was not detected in recordings from the pmCherry-R162W mutant transfected cells (Fig. 2B, circle trace).

Several prominent differences in current recordings were observed. Firstly, the current amplitude in all cells expressing the R162W channel was small. The inward current measured only 133±64 pA at −140 mV corresponding to an average current density of 8.35±3 pA/pF (n = 29). This response was just 25% of the wild-type channel current amplitude and was only slightly more than was found in non-transfected cells (63±7 pA at -140 mV, n = 5; Fig. 2B, triangle trace). The measured slope conductance for the mutant channel was 1.13 nS for currents between −50 mV to −130 mV, and 1 nS for currents between −30 to +10 mV. Secondly, the IV plot was ohmic (a straight line crossing through zero on the voltage axis) as shown in the Fig. 2C, which represents a normalized current voltage plot. Lastly, the R162W channel expressing cells were depolarized compared to the wild-type transfected cells, as confirmed by the plot of normalized current as a function of the applied voltage pulse (Fig. 2C). The cells were depolarized by ∼52 mV, from −64±2.4 mV (n = 9, hKir7.1) to −12±2.1 mV (n = 29, R162W), p<0.005. These results show that the mutant channel does not function in a way that would be expected for the normal Kir7.1.

Next, we tested the effect of a Kir7.1 channel blocker (Cs^+^) on the function of the mutant channel. Fig. 2D illustrates that in a 20 mM Cs^+^ bath solution, both inward and outward currents were reduced to amplitudes similar to recordings obtained from non-transfected cells. The current amplitude was reduced from 391±12 to 53±10 pA (n = 7) when measured at −150 mV. When current in the presence of Cs^+^ was subtracted from pretreatment values, the resultant I–V plot (Fig. 2D, triangle trace) of the Cs^+^ sensitive component did not differ from the pretreatment I–V plot (Fig. 2D, square trace). This result is consistent with the knowledge that CHO cells do not express native Kir7.1 channels [12]. The same experiment performed using pmCherry-R162W transfected cells showed that bath Cs^+^ had no significant effect on the mutant channel current. The current amplitude measured at −140 mV was only partially reduced from 133±64 pA to 109±31 pA (n = 5) with bath Cs^+^. In contrast to the wild-type channel, the calculated Cs^+^ sensitive current suggests that there is very little Kir 7.1 channel function.

### Mutant Channel Shows no Selectivity for Rb^+^

**Figure 3:**
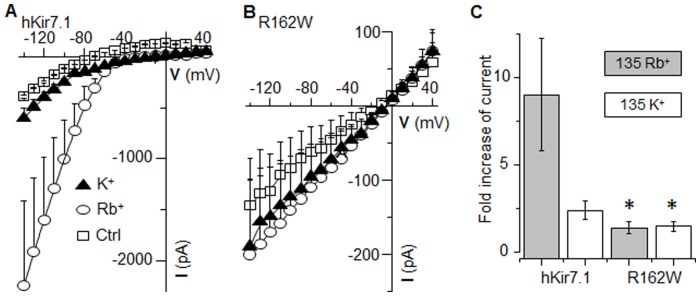
Rb^+^ has no effect on Kir7.1 R162W. Average I–V plot of pEGFP-hKir7.1 (**A**) and pmCherry-R162W (**B**) transfected cells. The recordings were obtained in HEPES Ringer’s (Ctrl: open square), 135 mM extracellular K^+^ (closed triangle), or 135 mM extracellular Rb^+^ (open circle). Each data point is the mean ± the SEM of at least 5 experiments. (**C**) Comparison of the mean fold-increase in the current amplitude due to the exposure of cells to either 135 mM external Rb^+^ (gray bar) or 135 mM external K^+^ (white bar) measured at −140 mV. Error bars are ± SEM.

Another important characteristic of Kir7.1 channels is that, unlike other members of the Kir family, Kir7.1 channels show a higher preference for Rb^+^ conductance over that of K^+^ [21]. Since the rat Kir7.1 mutant clone (R162W) was previously shown to form non-selective channels [1], we tested the mutant human channel for K^+^ and Rb^+^ selectivity (Fig. 3). As shown in Fig. 3A, pEGFP-hKir7.1 channel-expressing cells responded with a huge inward current in response to Rb^+^ in the bath (Fig. 3A). The current amplitude in response to a −140 mV voltage pulse increased from 391±12 pA in the presence of 5 mM extracellular K^+^ to 2247±601 pA in the presence of 135 mM extracellular Rb^+^ (n = 5). The results with the Rb^+^ bath solution showed at least a six-fold increase in the slope conductance of the inward current (5.4 to 33 nS, Fig. 3C), depolarization of the membrane potential, and substantially reduced amplitude of the outward current. Application of high extracellular K^+^ (Fig. 3A, triangle trace) solution resulted only in the depolarization shift of the membrane potential. A slight reduction in the slope conductance of the inward current (2.6 nS for high K^+^ vs. 5.4 nS for 5 mM K^+^) was also noticed. The current amplitude measured at −140 mV with high extracellular K^+^ was 601±94 pA (n = 5). Compared to the wild-type channel response, neither extracellular Rb^+^ nor K^+^ impacted current amplitudes due to the R162W channel (Fig. 3B and 3C). While Rb^+^ caused an average six-fold increase of current amplitude at −140 mV for the hKir7.1 channel, only a 1.5 fold increase was registered for the R162W channel. In pmCherry-R162W transfected cells, Rb^+^ increased current from 117±57 to 186±92 pA, while external high [K^+^] caused no significant increase in current (117±57 pA in 5 mM external K^+^ vs. 156±73 pA in 135 mM external K^+^, p = 0.112) amplitude. Overall, there was no significant difference between the effects of the two ions (K^+^ and Rb^+^) on R162W current.

### Coexpression of R162W Mutant with Kir7.1 Wild Type Alters I_Kir7.1_

**Figure 4:**
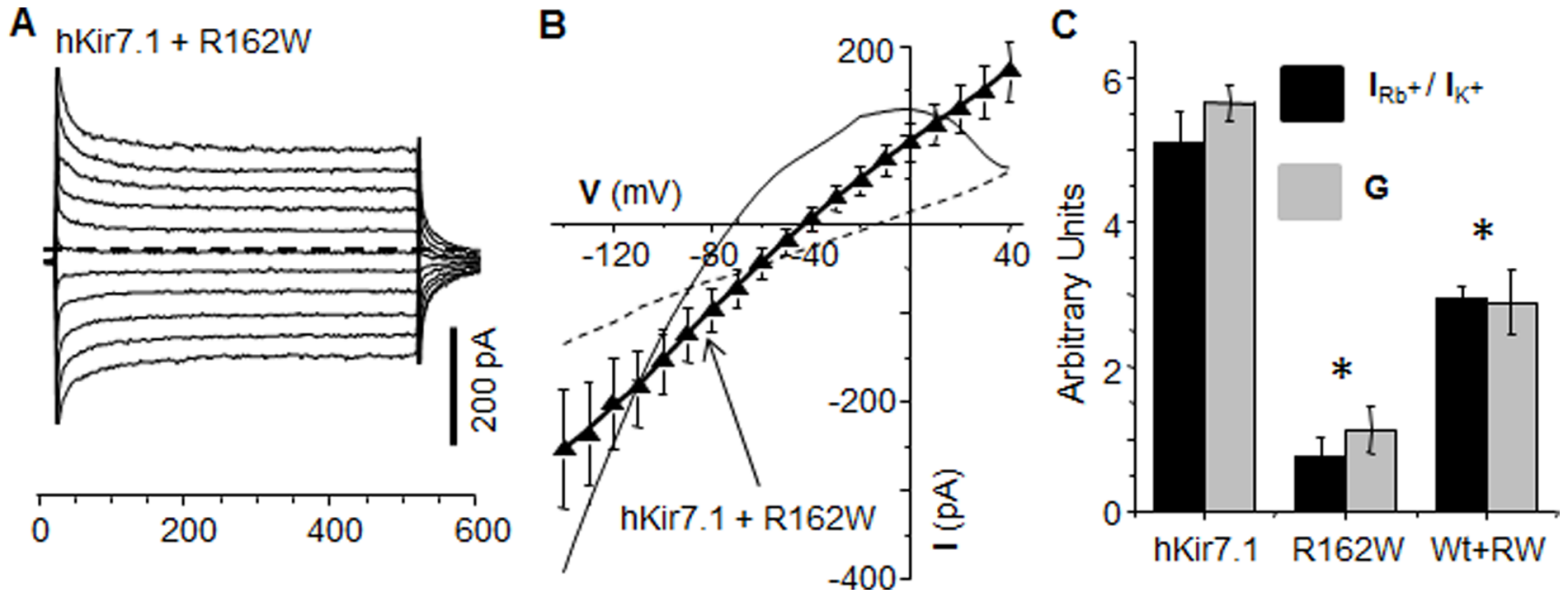
Mutation R162W affects the function of the Kir7.1 channel. Currents were elicited in cells co-transfected with equal amounts of pEGFP-hKir7.1 and pmCherry-R162W DNA during 500 msec 20 mV step voltage pulses from −160 to +40 mV. (**A**) Raw data of current recordings showing that responses were of similar amplitude in both ‘–ve’ and ‘+ve’ voltages. The dashed line represents zero current. (**B**) I–V relationship from seven co-transfected cells (solid triangle) shows a linear response. For comparison, the responses of hKir7.1 and R162W channel are shown as a solid line and a dashed line, respectively. Note that the current responses lacked rectification and that the zero current potential was intermediate between that of the wild-type and the mutant channel response. (**C**) Suppression of Kir current by the R162W mutation is illustrated by comparing the relative preference for Rb^+^ over K^+^ current responses at −150 mV (**I**_Rb_^+^/**I**_K_^+^: black bar) and the measure of the conductance of the inward current between −50 to −130 mV (**G**: gray bar). Mean values ± SEM from at least 5 experiments are represented.

The inheritance mechanism of SVD is autosomal dominant suggesting that co-expression of normal and mutant channel subunits results in a dysfunctional Kir channel. Thus, we co-transfected CHO cells with an equal ratio of wild-type and mutant plasmid (1 µg each per 35 mm dish) and studied the electrophysiological outcome. Current amplitudes showed step-wise increases and decreases in response to depolarizing and hyperpolarizing test voltage pulse potentials, respectively (Fig. 4A). The average current amplitude at −140 mV for the co-transfected cells was 260±66 pA (n = 10), significantly lower than the current amplitude recorded from the wild-type channel expressing cells (P<0.05). The steady state current-voltage plot for cells expressing both clones was linear (Fig. 4B) with an average resting membrane potential of -40±4.2 mV, and demonstrated a 24 mV depolarizing shift when compared to cells transfected with only the wild-type clone.

Measurement of the current density and Rb^+^ current also showed significantly reduced responses for the co-transfected cells compared to the wild-type channel transfection alone (P<0.001). Slope conductance is an accurate measure of the shape of the I–V plot. Positive slope conductance measured 2.89 nS and negative slope conductance measured 2.07 nS for the wild-type plus mutant channel expressing cells. Current amplitudes measured at −140 mV in the presence of Rb^+^, High K^+^, and Cs^+^ were 759±302 pA, 414±130 pA, and 221±86 pA, respectively. The average increase in current amplitude was only 3.1 fold for Rb^+^ (n = 8, P<0.0005) and 1.8 fold for high K^+^ (P<0.5) when compared to wild-type channel expressing cells.

We measured the ratio of Rb^+^ to high K^+^ sensitivity (**I**_Rb_^+^_/_ **I**_K_^+^) to determine Kir7.1 functional channel. Comparison between wild-type or mutant alone versus co-transfection showed significant alterations in channel function (Fig. 4C black bar, P<0.05). Likewise, significant reductions in channel conductance (**G**) were found (Fig. 4C grey bar, P<0.05). With an assumed Kir7.1 channel open probability of 0.45 and a unitary current of 0.312, the total number of functional channel for wild-type was normal at 2849, but the mutant and the wild-type plus mutant expressing cells were lower at 961, and 1674 channels, respectively. These results clearly suggest that the SVD mutation exerts a negative effect on the function of the wild-type channel. Such effects are consistent with the finding of abnormal retinal phenotypes in heterozygous carriers.

**Figure 5:**
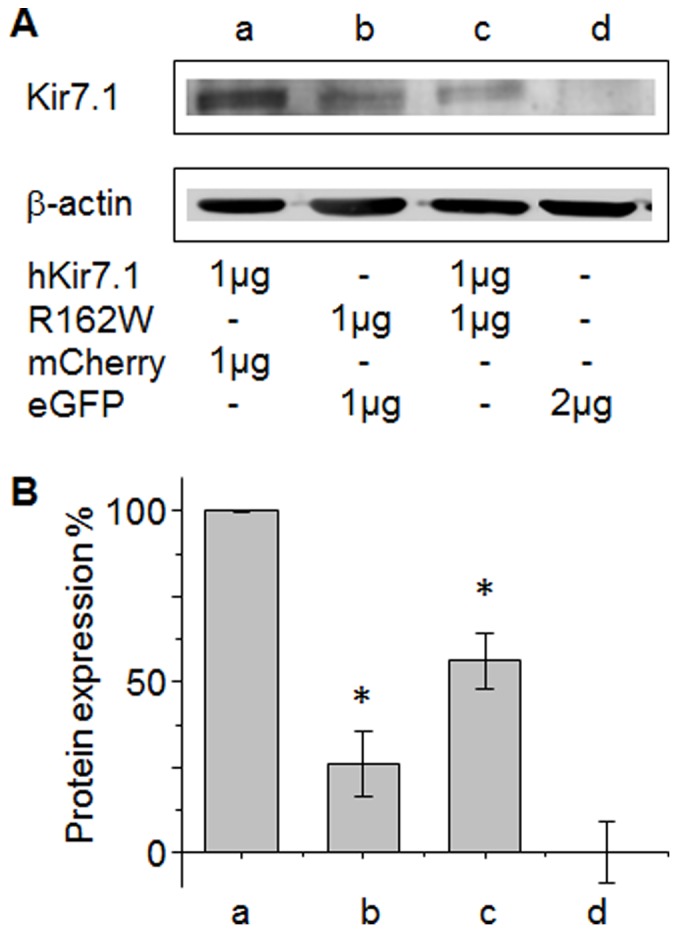
hKir7.1 and R162W protein expression in transfected CHO cells. (**A**) Western blot of transfected cell extracts with anti-Kir7.1 antibody showing Kir7.1 protein intensities at 54 kDa. The β-actin control confirms that consistent amounts of protein were loaded in each lane. The plasmids used for each transfection are indicated. (**B**) Total Kir7.1 levels were normalized against β-actin. The average total soluble Kir7.1 protein expression from the three different transfections used for the electrophysiology studies are presented as a ratio of protein expression to wild-type hKir7.1 protein levels.

The effect of the mutant channel on wild-type function may be a result of aberrant protein expression or localization. We performed Western blot analysis to assess protein expression. hKir7.1 wild-type and R162W mutant channels were transfected into CHO cells using equal DNA amounts (Fig. 5). Kir7.1 levels were equalized to β-actin and then all experiments were normalized to Kir7.1 wild-type only transfections to account for background intensities from blot to blot. Kir7.1 R162W expressing cells showed a 70% reduction in total Kir7.1 protein. When both Kir7.1 wild-type and R162W mutant were co-transfected, there was a 50% reduction in soluble total Kir7.1 when compared to the Kir7.1 wild-type. This suggests that there may have been a significant loss of the wild type channel due to oligomerization with mutant subunits which may have affected the assembly of a functional complex that would not pass the ER quality control machinery. Although co-transfection of wild-type and R162W plasmids increased the total soluble Kir7.1 protein fraction, it did not correct the mutant electrophysiology. Thus, we suggest that the incorporation of any R162W subunits within the tetramer alters Kir7.1 channel function.

### Membrane Localization of Mutant Kir7.1 Channel

**Figure 6:**
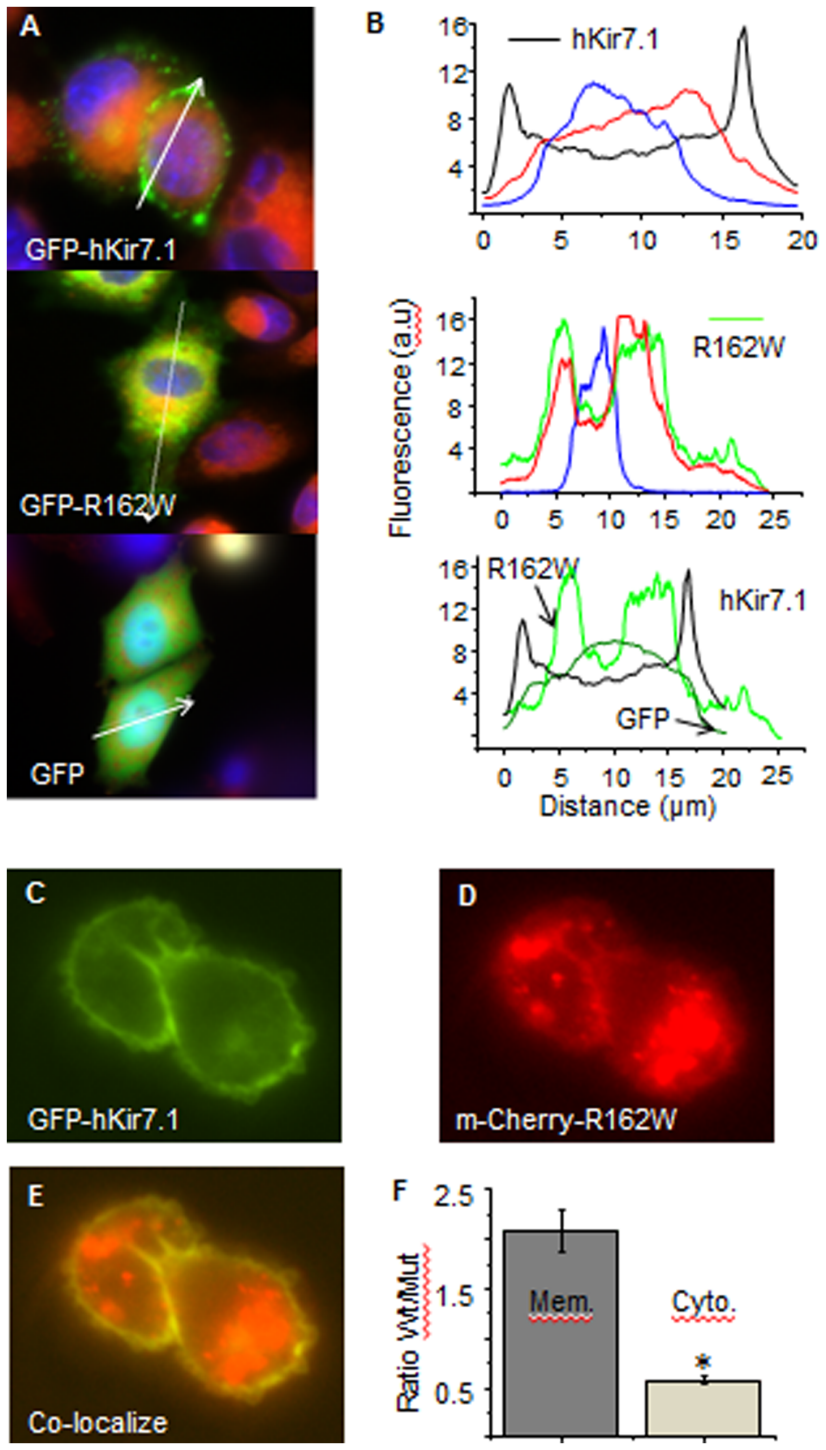
Cellular localization of the Kir7.1 channel. **CHO** cells expressing the pEGFP-hKir7.1, pEGFP-R162W (**A**) or both pEGFP-hKir7.1+ pmCherry-R162W (**C**, **D**, **E**) were studied by live cell fluorescence microscopy using a 60X water immersion objective. Kir7.1 localized mainly to the plasma membrane (**A**. upper panel green: hKir7.1, red: ER and blue: nucleus) in the pEGFP-hKir7.1 transfected cells. pEGFP-R162W expression co-localized with ER labeling (**A**. middle panel). Control pEGFP expressing cells are shown in the lower panel (**A**). (**B**) Line scans (white arrow) of fluorescence intensity distribution of pEGFP-hKir7.1 (**A**. black trace upper panel), pEGFP-R162W (**A**. green trace middle panel), and pEGFP (**A**. dark green trace lower panel) transfected cells. Red and blue traces (**B**. upper panel and middle panel) represent ER labeling and Hoechst nucleus staining, respectively. In co-transfection experiments, the GFP fluorescence localized to the cellular membrane (**C**), whereas mCherry fluorescence shows an intracellular aggregated localization (**D**). Superposition of both red and green fluorescence (**E**) further illustrates that there is very little co-localization of the wild-type and mutant channel signals. (**F**) Fluorescence quantification of membrane vs. cytoplasmic expression from five independent co-transfections with pEGFP-hKir7.1 and pmCherry-R162W plasmids is shown in **F**, p<0.01.

A loss of function of a channel protein occurs when there is altered trafficking of the protein to the membrane. To determine whether the SVD mutation alters the membrane localization of mutant Kir7.1 protein, we determined the relative fluorescence expression of the corresponding EGFP and mCherry signal. For cells transfected with pEGFP-hKir7.1, GFP fluorescence co-localized with the cell membrane (Fig. 6A top panel) and there was no overlap with either the ER or nuclear staining. This is depicted in the corresponding plot of the line scan (Fig. 6B (top panel) where Kir7.1 distribution is found on either end of the line scan (Fig. 6B top panel black trace), with no superimposition over the nuclear (blue) or ER (red) signal. In Fig. 6A (middle panel), a pEGFP-R162W expressing cell shows no green fluorescence at the membrane, but does show possible ER localization of the mutant protein. The line scan plot (Fig. 6B middle panel), shows GFP signal (green trace) overlapping the distribution of the ER signal (red trace) but not the distribution of nuclei (blue trace). GFP transfection alone (Fig. 6A, bottom panel), shows the expected distribution of green fluorescence throughout the cell, including nuclear localization. Line scans of Fig. 6A images further confirm membrane localization of the wild type protein (Fig. 6B lower panel black trace), ER localization of the mutant protein (Fig. 6E lower panel light green trace), and GFP localization within the cell (Fig. 6E lower panel dark green trace). Calculation of the average area under the curve for pEGFP-hKir7.1 (1154176±56700 µm^2^) was not significantly different from that of pEGFP-R162W (1115493±79990 µm^2^) indicating comparable levels of transfection. In cells co-transfected with pEGFP-hKir7.1 and pmCherry-R162W, EGFP fluorescence localized primarily to the cell periphery as expected when there is membrane localization of the wild-type channel (Fig. 6C). In contrast, mCherry fluorescence was predominantly visualized in the intracellular compartments (Fig. 6D), and was associated with a scattered perinuclear pattern of expression consistent with an ER distribution. Superimposed images of both green and red channels further demonstrated that there is a distinct localization of the wild-type channel within the membrane as opposed to the more perinuclear distribution of the mutant channel (Fig. 6E). Quantitative measurement of the fluorescence pixel value ratios between the membrane and cytoplasmic locations (Fig. 6F) confirmed significantly different distribution patterns of the wild-type vs. mutant channel in co-transfected cells (Fig. 6F, P<0.05).

### Homology Modeling Analysis of the Effect of the Substitution of Arg162 with Tryptophan

For Kir channels, an extracellular GYG conserved loop domain is implicated as the selectivity filter [2], but the recently published structure of Kirbac3 (bacterial Kir channel) protein also supports a regulatory role on K^+^ selectivity and permeation by the cytoplasmic domain [2], [22]. In particular, the C-terminal sequence immediately following the second trans-membrane (TM2) sequence has a group of conserved basic residues that participate in electrostatic interactions and provide flexibility in the C-linker domain which regulates the opening and closing of the channel [2]. These charged residues are in close proximity to the inner plasma membrane leaflet where binding sites for regulatory molecules like PIP_2_ are abundant [23].

**Figure 7:**
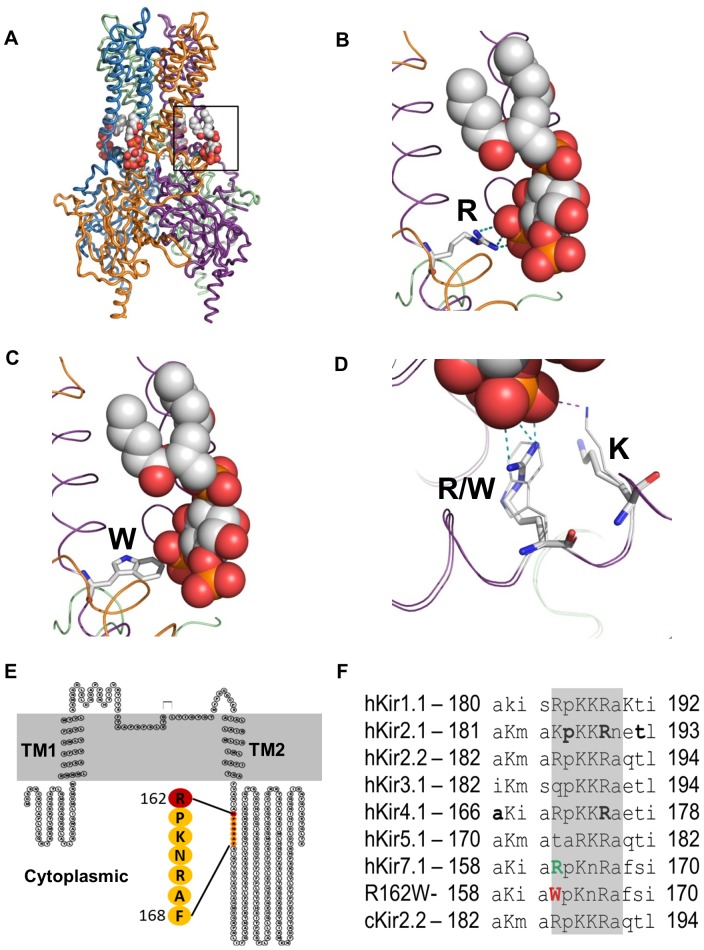
Human Kir7.1 model and Kir channel family homology within the C-linker domain. (**A**) Tetrameric structural model of Kir7.1 protein and four interacting PIP_2_ molecules. The highlighted structure is enlarged for clarity of the interactions between the C-terminal hotspot and the PIP_2_ head group. (**B**) R162 interacts with PIP_2_ through 3 hydrogen bonds as shown by the green dotted lines. (**C**) R162W structure showing the tryptophan residue and its side chain orientation with respect to PIP_2_. (**D**) Comparison of the interaction of both R and W at position 162 with PIP_2_ (green dotted line), along with the adjacent K-sharing hydrogen bond (purple dotted line). (**E**) Topology of the Kir7.1 subunit showing the relative position of the C-linker and Arg (R) 162 residue located adjacent to 2nd trans-membrane domain. (**F)** The conserved basic residues amongst Kir channels are indicated by upper-case letters. Disease mutations are highlighted by bold-face letters. Residues in the C-linker region are shaded. Numbers represent the first and last residues in the corresponding sequence. The species, name and accession numbers for proteins used for this comparison were as follows: hKir1.1 NM_000220, hKir2.1 NM_010603, hKir2.2 GI: 23110982, hKir3.1 NM_002239, hKir4.1 NM_002241, hKir5.1 NM_018658, hKir7.1 NM_002242, and cKir2.2 GI: 118097849.

We have recently demonstrated that PIP_2_ interaction with the Kir7.1 channel is an important determinant of function in the RPE cells [24]. Figure 7 shows three dimensional structural models of wild-type Kir7.1 and the mutant R162W based on Kir2.2 and Kir2.2R186A templates, respectively, using MODELLER software [15]. The models suggest that the R162W mutation does not disrupt the overall folding of the protein monomer or the assembly of the tetramer. The location of R162 on the surface of the monomer makes disruptions in the folding of the R162W protein unlikely. Arginine and tryptophan are of similar dimensions, and in both our model and experiments, the position of their side-chains is within the PIP_2_ binding site, rather than the monomer-monomer interface. The R162W mutation thus seems unlikely to disrupt the monomer-monomer interface.

Substitution of tryptophan results in the removal of a positively charged residue from the active PIP_2_ binding site. Figure 7B illustrates how the positioning of the positively charged arginine allows multiple hydrogen bonds with one of the PIP_2_ phosphate groups (green dotted lines). The tryptophan residue substitution removes these polar nitrogens from the binding site, making it less favorable for interaction with PIP2 and more favorable for interaction with the lipid membrane (Fig. 7C). The lysine at nearby position 164 might compensate somewhat for a missing arginine, but with a weakened interaction (Fig. 7D red dotted line). The net result of the R162W mutation appears to be a loss of strong non-covalent interactions with phosphate groups of the Kir7.1-PIP_2_.

Our homology modeling supports the hypothesis that R162W results in the loss of interaction with PIP2_,_ resulting in the loss of channel function. Further studies using molecular dynamics simulations are needed to confirm this result especially because aromatic residues can interact with lipid bilayers in multiple ways that impact folding and association [25], [26].

## Discussion

The RPE is a polarized epithelium with a distinct apical microvillar structure interdigitating with the photoreceptor outer segments. KCNJ13 gene expression in the RPE is in 19-fold excess when compared to its expression in the photoreceptors or choroid [27]. Consistent with this, we found Kir7.1 protein prominently localized to the RPE apical structures. We did not detect Kir7.1 protein in other parts of the retina [7], [28]. We confirmed our results using non-human primate, adult human, and fetal human retina preparations. Although Kir7.1 channels are also found in other organs, including brain and kidney [21], [29]–[32], the function of Kir7.1 channels has been studied only in RPE cells [7], [16], [17], [20], [33], [34].

In this paper, we show that expression of the wild-type human Kir7.1 channel in CHO cells results in a highly selective current, whereas expression of the human mutant Kir7.1 R162W resulted in current recordings that were linear, cells were depolarized, and the amplitude of the inward current was substantially reduced. Thus, the human Kir7.1 mutant channels are non-functional as a homo-tetramer but results in a dysfunctional channel when mutant channels are included in the hetero-tetrameric structure with wild type protein. A mutant dysfunctional channel was previously reported using a GFP fused rat Kir7.1 clone which formed a non-selective leaky channel that depolarized the cells [1]. Our findings cannot be attributed to altered function due to the GFP-fusion construct, as non-fusion versions of the Kir7.1 plasmid also resulted in a non-functional channel for mutant protein (Figure S1 in File S1). The rat Kir7.1 clone differed from our human clone in that it carried an additional mutation of A139S, which is also found in the wild-type rat [1]. In our study, the human Kir7.1 channel did not carry the A139S mutation so any additional effects due to this mutation can be parsed out. We have also shown that the human R162W mutant Kir7.1 channel depolarized the resting membrane potential to near zero with no preference for either K^+^ or Rb^+^. These results confirm the effect of this cytoplasmic mutation on causing a non-functional channel.

While Selectivity of the Kir7.1 channel is generally conferred by the selectivity P-loop located between the two trans-membrane domains on the external cell membrane [2] its gating is controlled by cytoplasmic domains. The unusual Rb^+^ selectivity of the Kir7.1 channel has been attributed to a Methionine (M) residue at position 125 within the selectivity P-loop, which has a conserved Arginine (R) in all other Kir channels [29]. For the Kir1.1 channel, a mutation of R128 to M results in a five-fold increase in Rb^+^ conductance over K^+^ [35]. Since the Kir7.1 residue R162 is located proximate to the cytoplasmic leaflet, there is a remote possibility that the interaction between the selectivity sequence and the R162 domain is altered by this mutation, thereby altering the Rb^+^ and K^+^ selectivity rather than due to a non-functional channel.

### Cytoplasmic C-linker Sequence Regulates Kir7.1 Channel Function

Several recent reports on Kir channel structure point to a possible role for the cytoplasmic C-linker sequence in determining channel selectivity [14], [22]. R162W is located within the C-linker structure (Fig. 7E), which is conserved among Kir channels (Fig. 7F). Our structural modeling results suggest that the R162W mutation alters several H-bonds. Since C-linker structures contribute to the flexibility of the protein structure and help in the twist and shrink motion [14], [22], a loss of flexibility due to the insertion of a non-polar tryptophan creates a stiffness in the structure possibly affecting controlled opening and closing.

A non-functional channel could be the result of the structural changes affecting its Golgi-to-plasma membrane trafficking. In this study, we have shown that the R162W mutant protein does not traffic to the membrane. For Kir2.1 channel Golgi-to-plasma membrane trafficking sequence is as associated with residues 242 to 246 [36]. Arginine possess two exposed sites for N-methylation, a post-translational protein modification that likely contributes to protein trafficking. A tryptophan residue at the same location will lack this modification. Zhang and colleagues recently reported that the R162W mutant protein is non-functional using an oocyte expression system [18]. In the Zhang study, mutant Kir7.1 channels successfully localized to both the Oocyte and Madin-Darby Canine Kidney (MDCK) cell membrane, whereas in our mammalian expression system, they do not. One explanation for this difference in our findings is that while the oocyte system is ideal for studying the physiology of ion-channels, it may not be effective in studying trafficking of channels and transporters due to a difference in culture temperature [37]. MDCK studies showed mutant channels with reduced localization to the membrane as compared to the wild-type channel, with the mutant channels confined to portions of the polarized membrane [18]. ER localization in the MDCK cells, if any, is unclear. MDCK cells are polarized epithelial cells that exclusively use sorting mechanisms for membrane expression of proteins. In the RPE cells, Kir7.1 channels are present only in the apical membrane. Directed trafficking of Kir7.1 channels is likely controlled through signals located within the extreme C-terminal domain, and it remains to be elucidated how this domain interacts with the R162 residue [11]. In the MDCK cells, no such polarized distribution of Kir7.1 channels was observed by Zhang *et. al.* upon transfection [18]. This suggests that channel trafficking in MDCK cells occurs irrespective of cellular sorting machinery. Our results are different because we used fluorescence fusion probes in live-cell imaging which is ideal for the biological imaging of proteins such as the Kir7.1 channel in living cells, and is less prone to experimental artifact. We have further shown in cultured human fetal RPE cells that co-transfection of wild-type and mutant channels had comparable results to that found in CHO cells (Figure S2 in File S1). We plan to determine the precise localization of the mutant protein in a future study using multi-pronged approach including cell-surface biotinylation, FRET, and spinning disc confocal microscopy with the use of a polarized RPE cell culture.

### Residue 162 is Involved in PIP_2_ Regulation

Another important role of arginine at position 162 relates to the interaction with PIP_2_ in the inner leaflet. All Kir channels are activated by PIP_2_ [23], [38], and one of the PIP_2_ binding sites maps to a cytoplasmic hotspot ‘bPbbb’ (b-basic residue, and P-proline), as illustrated in Fig. 7 (E and F) [39], [40]. A basic residue in position 162 is conserved in all Kir channels and participates in both the PIP_2_ and channel interactions (Fig. 7F). As reviewed recently by our group and summarized in Fig. 7F, Kir channel mutations within the hotspot have been associated with several disease phenotypes [39]. PIP_2_ regulates the function of the Kir7.1 channel in the RPE [24], stabilizes the Kir structure in the membrane [14], and also helps in the posttranslational assembly and trafficking of the channel to the membrane [41].

The Kir channel is a tetrameric protein and PIP_2_ molecules interact with the binding site interfacing between two adjacent subunits [14], [42], [43]. It remains to be determined how mutant subunits might be incorporated into the tetrameric protein structure. For the Kir2.1 channel, the alteration of a single PIP_2_ binding site has been suggested to alter channel function [42]. Our modeling analysis in this study is based on the recently solved Kir protein structure including PIP_2_ molecular interactions [14], [22]. Zhang *et. al.* recently inferred that R162W alters the Kir7.1 channel-PIP_2_ interaction [18], but our results suggest the physical mechanism by which this occurs. It is likely that the mutant R162W prevents both PIP_2_ binding, which may then prevent translation of the cytoplasmic domain towards the membrane. Also based on the location of R186 in Kir2.2, our homology model of Kir7.1 suggests that R162W would contribute to determining the conductance and selectivity of the Kir channel as reported previously [22]. Homology modeling as in here however does not predict actual change in the amino- acid side-chain interaction with the environment (solvent and/or lipids). It will be of interest to determine if mutations at position 162 in other Kir channel family members also result in non-functional and/or non-selective channels.

### Kir7.1 Defect Explains Classical SVD Phenotype

Various SVD clinical features, including snowflake depositions, focal RPE degeneration, and moderate myopia could be the result of a non-functional apical membrane Kir7.1-channel that leads to altered RPE physiology. Kir7.1 conductance contributes to the hyperpolarized RPE apical membrane potential that aids in RPE/Photoreceptor communication [44], [45]. Blocking Kir7.1 channel function can depolarize the RPE apical membrane [24], [33], [34], [46] but will also affect the basal membrane potential [46]. In the case of SVD, depolarization of the RPE membranes as shown by co-expression of wild-type and mutant Kir7.1 channels could affect the photoreceptor-RPE interaction and compromise the integrity of the epithelial barrier thereby contributing to the snowflake-like depositions that are ultimately associated with RPE degeneration.

The myopic eye is characterized by an increased axial dimension and a large vitreal volume. Crewther and colleagues demonstrated in chicks that RPE Kir7.1 channels work together with the apical membrane NKCC co-transporter to regulate refractive compensation [47]. Inhibition of the Kir7.1 channel by Ba^2+^ does not compensate for the imposed optical defocus [47]. This finding supports our hypothesis that the vitreal volume increase is a result of decreased fluid flow across the RPE [48]. Both myopia and the liquid vitreous, findings reported in SVD patients, might thus arise from changes in fluid flow across the RPE.

### Sub-retinal K^+^ Homeostasis is an Important Contributor to ERG

We propose that the abnormal ERG b-wave in SVD patients is due to a disturbance in the ionic environment of the sub-retinal space which affects the net spatial K^+^ buffering across the retina [49]. The sub-retinal space experiences a light-evoked decrease in K^+^ concentration that partially hyperpolarizes both the Müller cells and the photoreceptors. A drop in the sub-retinal space K^+^ will also depolarize RPE cells by a change in the trans-membrane potential. In the RPE apical membrane, potassium is continuously recycled via Na^+^-K^+^-ATPase, which aids in the uptake of K^+^, whereas Kir7.1 helps in K^+^ efflux to the sub-retinal space. One of the key events following light exposure is the release of a light peak substance from the retina that activates a RPE G-protein Coupled Receptor (GPCR). Since Kir7.1 channels are activated by PIP_2_, activation of a light-induced GPCR will inhibit the Kir7.1 channel and result in the depolarization of the RPE apical membrane. Subsequently, re-synthesis of PIP_2_ occurs to ensure Kir channel reopening to reactivate channels and to restore the sub-retinal space K^+^ to resting levels. A mutant Kir7.1 is non-functional, and hence is non-responsive to the normal light-induced regulations. The b-wave originates at least in part from the on-bipolar cell depolarization that occurs in response to the light-induced reduction in glutamate-release from the photoreceptors. Gross changes in the resting membrane potential of both the photoreceptors and the Müller cells are expected due to changes in the sub-retinal space K^+^ homeostasis mechanisms and likely contribute to the b-wave abnormality seen in SVD patients.

In summary, we have shown that the Kir7.1 R162W mutation that is associated with SVD forms a non-functional channel when expressed in a heterologous system. Since the Kir7.1 channel is tetrameric, the underlying mechanism of disease is likely the result of the inclusion of mutant subunit(s) in the assembly process that alter the structure and function of the resultant channel. Kir7.1 mutations have also been associated with Leber’s Congenital Amaurosis (LCA), which, together with the SVD findings, suggests that this ion channel plays an important role in eye disease [50], and by extrapolation, in diseases associated with other organ systems where Kir7.1 channels may influence tissue function [51].

## Supporting Information

File S1 Contains Figure S1 and Figure S2.
(PPTX)
