# Supplementary material for: Snowflake Vitreoretinal Degeneration (SVD) Mutation R162W Provides New Insights into Kir7.1 Ion Channel Structure and Function
Source: PLoS One. 2013 Aug 19;8(8):e71744. doi: 10.1371/journal.pone.0071744 (PMC3747230; doi:10.1371/journal.pone.0071744)
Supplement: File S1 — Contains Figure S1 and Figure S2. (PPTX) [file pone.0071744.s001.pptx]

## Slide 1
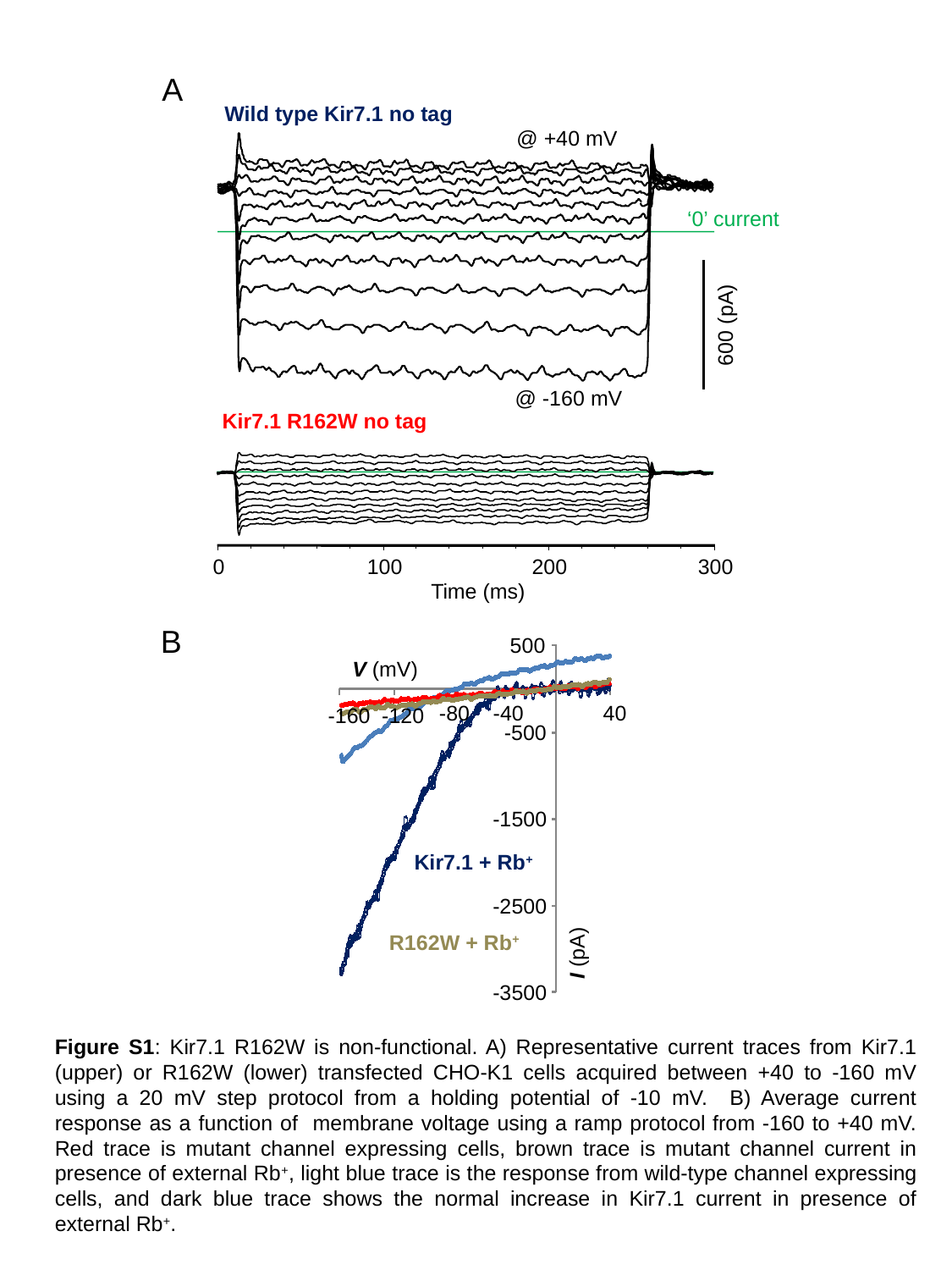

A
Wild type Kir7.1 no tag
@ +40 mV
‘0’ current
600 (pA)
@ -160 mV
Kir7.1 R162W no tag
0
100
200
300
Time (ms)
B
500
V (mV)
-80
-40
40
-160
-120
-500
-1500
Kir7.1 + Rb+
-2500
R162W + Rb+
I (pA)
-3500
Figure S1: Kir7.1 R162W is non-functional. A) Representative current traces from Kir7.1 (upper) or R162W (lower) transfected CHO-K1 cells acquired between +40 to -160 mV using a 20 mV step protocol from a holding potential of -10 mV. B) Average current response as a function of membrane voltage using a ramp protocol from -160 to +40 mV. Red trace is mutant channel expressing cells, brown trace is mutant channel current in presence of external Rb+, light blue trace is the response from wild-type channel expressing cells, and dark blue trace shows the normal increase in Kir7.1 current in presence of external Rb+.

## Slide 2
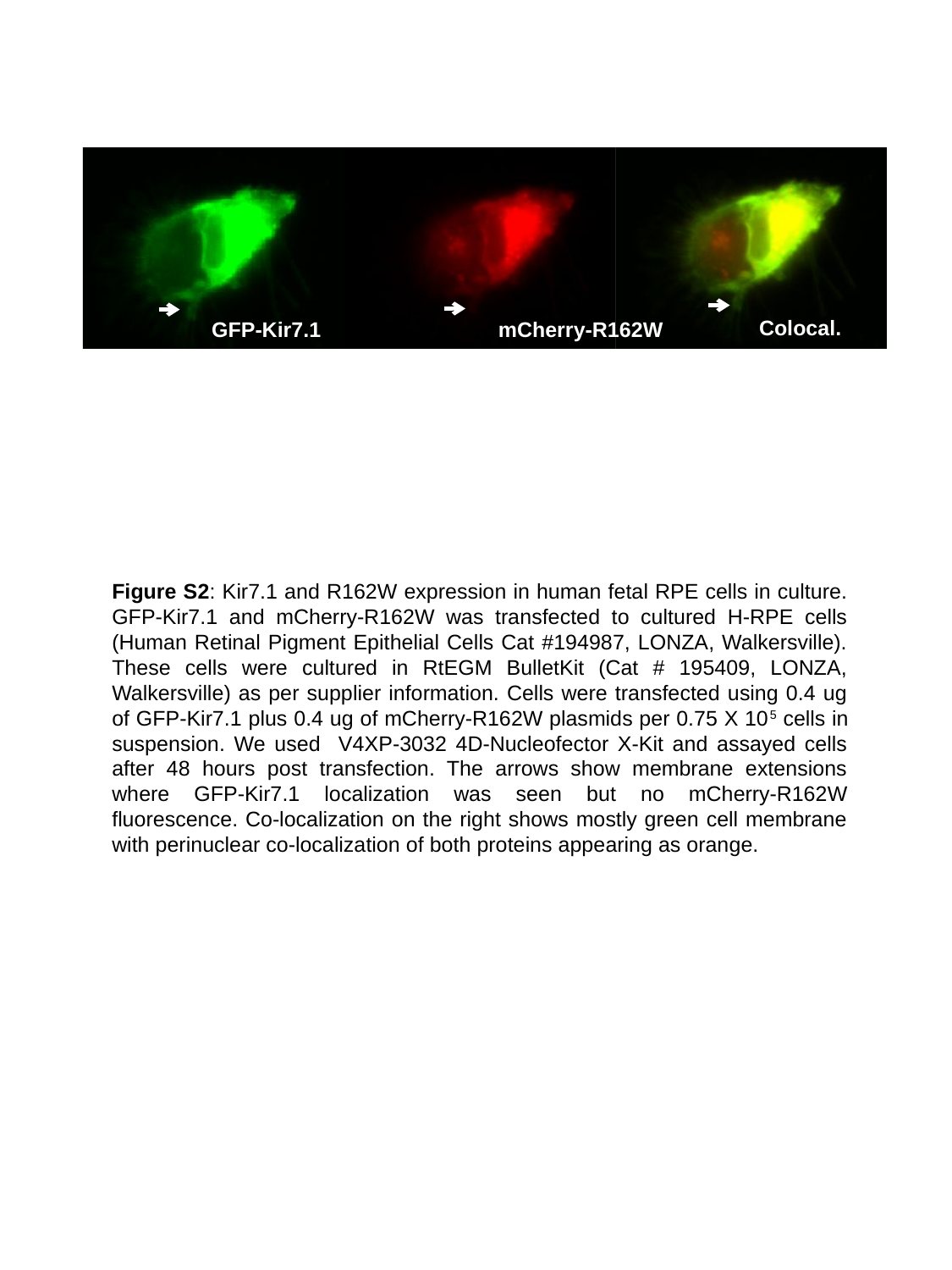

Colocal.
mCherry-R162W
GFP-Kir7.1
Figure S2: Kir7.1 and R162W expression in human fetal RPE cells in culture. GFP-Kir7.1 and mCherry-R162W was transfected to cultured H-RPE cells (Human Retinal Pigment Epithelial Cells Cat #194987, LONZA, Walkersville). These cells were cultured in RtEGM BulletKit (Cat # 195409, LONZA, Walkersville) as per supplier information. Cells were transfected using 0.4 ug of GFP-Kir7.1 plus 0.4 ug of mCherry-R162W plasmids per 0.75 X 105 cells in suspension. We used V4XP-3032 4D-Nucleofector X-Kit and assayed cells after 48 hours post transfection. The arrows show membrane extensions where GFP-Kir7.1 localization was seen but no mCherry-R162W fluorescence. Co-localization on the right shows mostly green cell membrane with perinuclear co-localization of both proteins appearing as orange.
